# Gbrowse Moby: a Web-based browser for BioMoby Services

**DOI:** 10.1186/1751-0473-1-4

**Published:** 2006-10-24

**Authors:** Mark Wilkinson

**Affiliations:** 1 Assistant Professor, Department of Medical Genetics, University of British Columbia, James Hogg iCAPTURE Centre for Cardiovascular and Pulmonary Research, St. Paul's Hospital, Rm. 166, 1081 Burrard St., Vancouver, BC, V6Z 1Y6, Canada

## Abstract

**Background:**

The BioMoby project aims to identify and deploy standards and conventions that aid in the discovery, execution, and pipelining of distributed bioinformatics Web Services. As of August, 2006, approximately 680 bioinformatics resources were available through the BioMoby interoperability platform. There are a variety of clients that can interact with BioMoby-style services. Here we describe a Web-based browser-style client – Gbrowse Moby – that allows users to discover and "surf" from one bioinformatics service to the next using a semantically-aided browsing interface.

**Results:**

Gbrowse Moby is a low-throughput, exploratory tool specifically aimed at non-informaticians. It provides a straightforward, minimal interface that enables a researcher to query the BioMoby Central web service registry for data retrieval or analytical tools of interest, and then select and execute their chosen tool with a single mouse-click. The data is preserved at each step, thus allowing the researcher to manually "click" the data from one service to the next, with the Gbrowse Moby application managing all data formatting and interface interpretation on their behalf. The path of manual exploration is preserved and can be downloaded for import into automated, high-throughput tools such as Taverna. Gbrowse Moby also includes a robust data rendering system to ensure that all new data-types that appear in the BioMoby registry can be properly displayed in the Web interface.

**Conclusion:**

Gbrowse Moby is a robust, yet facile entry point for both newcomers to the BioMoby interoperability project who wish to manually explore what is known about their data of interest, as well as experienced users who wish to observe the functionality of their analytical workflows prior to running them in a high-throughput environment.

## Background

The BioMoby Project [1-3] was initiated in late 2001 as an open-source initiative within the model organism database and partner community with the goal of identifying standards and/or conventions that would aid interoperability between the diverse bioinformatics resources currently available online. The BioMoby Web Service interoperability platform [4,5] is now used to expose more than 680 bioinformatics data and analytical resources throughout North America, Europe, and Asia, with participants from South America and Australia now beginning to come on-line.

The interest in BioMoby-based services stems from its ability to identify service providers that are capable of consuming a particular in-hand data-type, manipulating it in a particular way, and producing a well-defined output data-type that can then be reliably and automatically consumed by another service provider without any further manipulation. Most of these discovery/execution processes can be fully automated, thus releasing the end-user (biological researcher or bioinformatician) from the task of discovering the Web interface to their tool of interest, manually manipulating their data format to conform to that Web interface, and then copy/pasting the result from their browser.

The BioMoby interoperability system consists of:

• An ontology describing biological data entities (the Namespace ontology), for example, records from a particular database such as Protein Data Bank or GenBank.

• An ontology describing biological data structures (the Object ontology), such as FASTA or Generic Feature Format.

• An ontology describing bioinformatics service types (the Service ontology), such as BLAST or ClustalW

• A novel Web Service registry – Moby Central – that acts as a "yellow pages", employing these ontologies to semantically and syntactically match the needs of a service consumer with the appropriate service provider.

The interoperable behaviours observed between BioMoby service providers is achieved through all providers agreeing to use, or extend, the standard set of data entities and data structures defined in the Namespace and Object ontologies when designing their Web Services.

To facilitate the exploration of BioMoby-compliant services, a variety of client programs have been constructed. We present here the Gbrowse Moby client, a browser-style interface that enables the guided construction and stepwise execution of analytical workflows. This is achieved through iterative discovery of all relevant services available as the potential next step in the workflow, followed by execution of the selected service, and rendering of the results; result data can be automatically used to seed the next service discovery query. Gbrowse Moby was the first Web portal to facilitate one-stop, interoperable access to hundreds of uncoordinated databases and analytical tools, and remains one of the most frequently used gateways into the BioMoby data and analytical space. The Semantic Web Service browsing paradigm established by Gbrowse Moby represents a novel and powerful way of interacting with biological and bioinformatics data and services.

In the following, we will describe the most salient and visible features of this interface, as well as some less obvious features that allow it to be accessed by third-party tools such as the Gbrowse package from the Generic Model Organism Database project (GMOD) [6], or to be used transparently as a portal through easily-parsed Common Gateway Interface (CGI) GET calls to the public interface.

## Implementation

Gbrowse Moby is implemented as a Perl CGI script that can run under most Web servers. It requires a full installation of the BioMoby Perl code libraries [7], the Perl Life Sciences Identifier (LSID) resolution stack [8], and the Bio::Graphics::Browser::Util configuration module from the Gbrowse package of GMOD [9]. Though Gbrowse Moby can run entirely independently of the Gbrowse interface itself, the configuration required to link the Gbrowse on-screen widgets to the Gbrowse Moby interface is described in detail in the Gbrowse package documentation. Look-and-feel are modified by editing the Gbrowse configuration file such that Gbrowse Moby can have an identical appearance to Gbrowse itself.

## Results and discussion

### The search and execute interfaces

The Gbrowse Moby interface is designed to facilitate one particular style of interaction with the BioMoby registry and services – that is, the discovery of service providers based on a query of the MOBY Central registry focused on the particular type of input data (i.e. a combination of Namespace and/or Object types) that is in-hand at any given point in the exploration process. As such, a browsing session must first be primed with some input data. In Gbrowse Moby, this initializing data takes the form of an identifier for some type of database record. An initialization screen is shown as the introductory page to assist in selecting the database domain (Namespace) of the identifier – what type of record is being identified – and the ID number of that record. When the user clicks the "Explore" button, a search is executed against MOBY Central to discover services capable of providing more information about that particular database record.

To interpret the results of such a query, it is critical that users be aware of a fundamental principle of the BioMoby environment – that is, that a Namespace is not tied to a particular service provider. For example, the NCBI_gi (GenBank) Namespace can be utilized by *any *service provider who claims to be capable of providing information about a particular GenBank record, not just GenBank themselves. This foundational principle of the BioMoby platform allows third parties to provide additional information, of any type, for any given database record, and thus dramatically expands the amount and type of data that can be discovered through BioMoby exploration.

**Figure 1:**
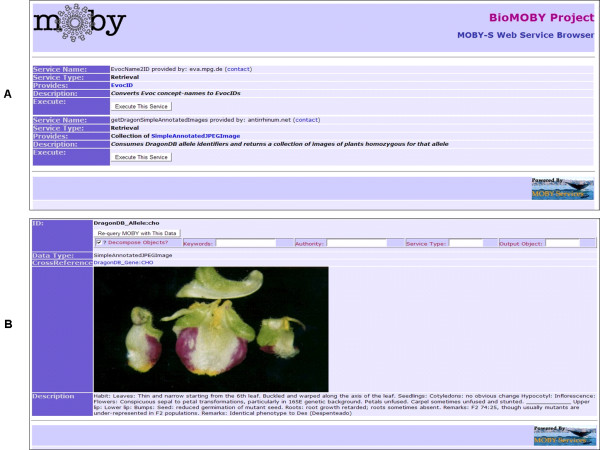
**The Search Result Screen (A) and the Invocation Result Screen (B) of the Gbrowse Moby Web Service Browser**. (A) shows two services discovered using the search parameters: Namespace = "DragonDB_Allele", Identifier = "cho". The latter of these, getDragonSimpleAnnotatedImages provided by antirrhinum.net, has been executed, with the results shown in panel (B). The rendering in (B) comes from a combination of two automatically selected renderers in response to the unknown data-type "SimpleAnnotatedJPEGImage"; one was capable of rendering JPEG Images, and the other capable of rendering free-text. Note also the clickable cross-reference to the DragonDB_Gene CHO in panel (B) that will initiate a new Gbrowse_Moby browsing session primed with that new piece of cross-referencing information provided by the Service.

After initialization, there are two displays that make up the Gbrowse Moby interface: the Service Search Result display, and the Service Invocation Result display. These are shown in Figure 1A, and 1B respectively. These can be configured to match the "look and feel" of the host website through a simple cascading style sheet and configuration file, based on the Gbrowse configuration file from GMOD.

The results of a search against the Moby Central web-service registry are presented as a list of compatible services in the Search Result screen (Fig 1A). Service descriptions include:

• The name of the service

• The identification of the service provider – their "Authority" identifier

• The service type ontology term – what type of analytical operation the service provides

• The ontologically defined data-type that will be returned from invocation of that service

• A human-readable description of what the service does, and what resources it uses

• An email link to contact the service provider if necessary

To execute a desired service using the most recently-displayed data as input, the user simply clicks the "Execute this Service" button. No additional formatting of the data is required, and the user does not need to be aware of any details about the interface for that service – all of these functions are managed by the Gbrowse Moby software.

If a service offers additional configuration parameters a "Configure Parameters" button is available in addition to the "Execute this Service" button. Parameter configuration happens thorough a JavaScript pop-up window, in which each parameter is presented with its default value pre-selected. Users can modify the default values if they require, and these are validated against the data-type and/or range that the service provider has specified in the registry prior to being saved. The modified values will be passed to the service when it is executed.

The Invocation Result screen (Fig. 1B) presents the results of the service execution rendered as HTML (the rendering process is described in detail in the Data Rendering section). In the case of an error in service invocation, an error code (defined in the BioMoby Service API), plus the textual description of the error as provided by the service provider, are displayed. If the service provider provides no error code information, Gbrowse Moby makes a "best guess" at what the error situation is based on the portion (if any) of the response message it received from the service provider.

Each result is associated with a "Re-Query" button, and a set of query restriction parameter boxes. Unless additional parameters are chosen, clicking the "Re-Query" button executes a query against the Moby Central registry based on the data-type that is currently being displayed; i.e. the default query is "what services consume the data currently in-hand". This search can be further refined by the inclusion of one or more of:

• A desired service type, for example "Parsing" or "BLAST"

• A particular service provider, based on a unique Authority identifier

• A desired type of output data

• A keyword found in the service description

• A switch that enables matching of services that operate on semantically and syntactically compatible data-types

Output data from the previous service invocation is passed to whichever service is selected by clicking the "Execute This Service" button. Executing a service returns the user to the Invocation Screen, and the process iterates. Thus, the user is led through potentially lengthy and complex bioinformatics workflows by an interface that provides a uniform view over hundreds of Web resources, suggesting only those that are capable of operating on the data-type currently in-hand.

As the user continues their exploration of the data-space, Gbrowse Moby records a (linear) record of their activities. This record can be saved at the end of a browsing session by retrieving it as an XSCUFL (XML Simple Unified Conceptual Flow Language [10]) document by clicking the button labeled "Retrieve Current SCUFL Workflow". The resulting XSCUFL workflow is compatible with high-throughput workflow environments such as Taverna [11].

New resources appear in the interface as they are registered in the BioMoby registry, unlike other bioinformatics analysis tools where the data-types and interoperable resources are hard-coded into the interface. The breadth and scope of interoperable resources available through Moby, and thereby through the Gbrowse Moby browser, is limited only by the participation of the community.

### Data rendering

The most crucial consideration in the design of Gbrowse Moby was its data-rendering architecture. A significant complexity arises from the fact that the BioMoby Object (data-type) ontology is end-user extensible. This makes it possible, even likely, that data returned to the Gbrowse Moby client from any given service will be in a format never before encountered; nevertheless, the system must not fail when receiving these unpredictable data-types, since this unpredictability is a necessary consequence of the open-world BioMoby specification.

Gbrowse Moby employs a novel, ontology-guided rendering system that allows it to render any Moby data-type it receives. The rendering methodology is as follows:

• HTML renderers are assigned to, at a minimum, the base of each major "branch" of the Object ontology.

• At startup, Gbrowse Moby polls all available renderers for the Ontology nodes that they are capable of rendering.

• When a piece of data (i.e. an instance of an Ontology node) is received by Gbrowse Moby, it first checks if it has a renderer for that data-type.

◦ If so, that renderer is selected

◦ If not, it traverses the Object ontology until it discovers an ontological parent that has a renderer assigned to it, and that renderer is selected.

• The data is passed to the renderer

• The renderer disassembles the XML of that object, rendering only the XML nodes that it "understands", and removes them from the data model.

• The renderer then passes the HTML-rendered data back to Gbrowse Moby, along with the fragments of the data object that were left unrendered. ***Because all data in BioMoby is ontologically defined, each of these data fragments is itself an instance of an ontologically defined data-type.***

• Gbrowse Moby iterates through this process with each data fragment, eventually finding a renderer capable of rendering each fragment.

This rendering methodology has been robust over the hundreds of new data-types registered in the Object ontology in the past four years (24 new data-types registered in the month of July, 2006, alone) and thus we encourage others to follow this as a paradigm for dealing with the ever-expanding BioMoby Object Ontology.

A default set of renderers is available in the Gbrowse Moby package which is capable of rendering all existing MOBY objects at a superficial level. For richer or more graphical representations of certain Object types, new renderers can be created by the host with no modification of Gbrowse Moby itself. The API for the renderers consists of only two methods – "type" which returns the names of the ontology node(s) that the renderer can manage; and "render" which returns the HTML representing that Moby Object, and any un-rendered fragments.

### Invoking Gbrowse Moby with a CGI GET String

It is often desirable to initiate a BioMoby registry query, or invoke a BioMoby service, from inside of another application. For example, you may want to create hyperlinks to Gbrowse Moby Searches from a gene name displayed on a third-party web page, or allow a displayed sequence to be automatically passed to a particular sequence alignment service simply by clicking. To facilitate this, Gbrowse Moby can receive, as CGI GET string parameters, a service name, an authority, a Namespace, a data identifier, and/or a URL-encoded block of XML representing a piece of BioMoby data. This allows other standalone or Web-based applications to utilize the Gbrowse Moby interface without having to know anything about the BioMoby specification or have any of the BioMoby code libraries installed. This functionality is the means by which Gbrowse Moby is integrated with the Generic Genome Browser (Gbrowse) – the Gbrowse sequence viewer display can be configured such that individual features in the genome map are hyperlinked to a Gbrowse Moby browsing session primed with whatever sequence-feature the user clicked on, simply by passing the namespace and id of the selected feature as GET string parameters in the hyperlink.

It is also possible to utilize Gbrowse Moby without initiating the browser at all. If namespace, id, authority and service name are all passed in the GET string, the indicated service will be executed, and the rendered HTML result returned (see [12] for an example). To make it convenient to extract this data from within independent standalone applications, tags are placed into the rendered Gbrowse Moby HTML that facilitates the parsing individual service results. The HTML comment tags:

<!--SCRAPE_ME_START -->

<!--SCRAPE_ME_END -->

## Limitations

As a pure-CGI-based browser system, there are several limitations to Gbrowse Moby. The most notable of these is the way it handles BioMoby Collections. While BioMoby services are capable of consuming and/or producing collections of input or output data-types (suitable for high-throughput analyses) Gbrowse Moby is specifically intended to be a low-throughput manual browser. As such, output collections are broken into their individual components, and can only be passed singly to downstream services. Attempts have been made to provide support for Collections, however the scale of output data quickly becomes unmanageable within a browser-style client, so this functionality has been purposely removed in order to keep the Gbrowse Moby interface as simple as possible.

The utility of the GET string functionality is limited by the server-specific size-limitations on GET requests. For even moderately large data objects, the URL-encoded string would be larger than many HTTP servers will allow in a GET operation; as such, this functionality is best utilized only for simple invocations of services that require only namespace and id parameters.

## Example annotated workflow

A multi-step workflow representing a typical Gbrowse Moby browsing session is provided (see Table 1) that can be followed to observe the salient features of the interface. In this example, the user initializes the browsing session with the keyword "apetala3" (an *Arabidopsis thaliana *gene common-name). This triggers a semantic search against the MOBY Central Web Service registry for services that operate on keywords. Among the results, are a service offered by mips.gsf.de named GetAGILocusCodes, which returns Arabidopsis Genome Initiative (AGI) locus identifiers. Invocation of this service results in a single piece of output data, the AGI Locus At3g54340 – the formal identifier for the apetala3 gene. Reinitializing with that piece of data triggers a new semantic search, from which the getEmblDNASequence service can be selected to retrieve the DNA sequence for that locus, and so on through the remainder of the workflow.

**Table 1:** An annotated example Gbrowse Moby workflow

**Step**	**Screen**	**Data Type **or *Service (provider)*	**Notes**
1	Initialization	**Namespace: Global_keyword ID: apetala3**	*Select namespace and type in the ID*
2	Search	*GetAGILocusCodes (mips.gsf.de)*	
3	Invocation	**AGI_LocusCode:At3g54340**	
4	Search	*getEmblDNASequence (mips.gsf.de)*	
5	Invocation	**CommentedDNASequence**	*type FASTA into the desired output object field and Re-initialize*
6	Search	*GenericSequence2FASTA (bioinfo.icapture.ubc.ca)*	
7	Invocation	**FASTA**	*Switch the authority to antirrhinum.net to limit to the Snapdragon database. Switch semantic searching OFF to limit to services that specifically consume FASTA files.*
8	Search	*getDragonBlastText (antirrhinum.net)*	
9	Invocation	**NCBI_Blast_Text**	*keep the authority as antirrhinum.net and resubmit*
10	Search	*parseDragonDBBlastText (antirrhinum.net)*	
11	Invocation	**DragonDB_Sequence**	*Chose EM:AMDEFA*
12	Search	*getDragonSequenceLocus (antirrhinum.net)*	
13	Invocation	**DragonDB_Gene**	*Chose DEF*
14	Search	*getDragonLocusAlleles (antirrhinum.net)*	
15	Invocation	**DragonDB_Allele**	*Chose def-101*
16	Search	*getDragonSimpleAnnotatedImages (antirrhinum.net)*	
17	Invocation	**SimpleAnnotatedJPEGImage**	
			*traverse back (with browser back button) to AGI_LocusCode:At3g54340*
4a	Search	*getGOTermsByAGIcode (atidb.org)*	
4b	Invocation	**GO Term**	
4c	Search	*getGOTermAssociations (bioinfo.icapture.ubc.ca)*	
*4d*	Invocation	**GO Associations**	
			*traverse back (with browser back button) to AGI_LocusCode:At3g54340*
4aa	Search	*getNASC_codebyAGI_locus (arabidopsis.info)*	
*4ab*	Invocation	**NASC_Code**	*note all of the cross-references, all clickable to reinitialize the browsing session*
			*traverse back (with browser back button) to AGI_LocusCode:At3g54340*
4aaa	Search	*getAGRISTFFamilyNameByAGI (arabidopsis.med.ohio-state.edu)*	
*4aab*	Invocation	**AGRIS Transcription Factor Family Name**	

Steps in the browsing session are enumerated, sequentially. Numbers with suffixes (e.g. 4a) indicate an alternate path (a) starting from step 4. The column labeled Screen indicates which Gbrowse Moby screen is visible at any given step. The middle column indicates which Moby Service (for the Search screen) should be selected, or which output data-type (for the Initialization and Invocation screens) is being displayed on the screen. The Notes column provides browsing hints and/or observations that can be made at any given step.

Of particular note are the ability to limit searches based on output data-type (Step 5 of the sample workflow); to broaden or narrow your search by applying/removing ontology traversal during the interpretation of the query data-type (Step 7); the ability to limit searches to specific service providers (Steps 7 and 9); and the ability to follow cross-referencing information (Step 4ab).

An example of the iterative rendering process is exhibited in Step 17 of the sample workflow. Gbrowse Moby includes a renderer for base-64-encoded JPEG images, and a renderer for Strings; however it does not have a renderer for the SimpleAnnotatedJPEGImage data-type. Examination of the ontological relationships that make up SimpleAnnotatedJPEGImage (see Appendix 1) shows that it is a type of base-64-encoded-JPEG that contains an additional String data-type representing the description. In this case, the rendering system queried the ontology for the parentage of SimpleAnnotatedJPEGImage, discovered it was a type of base-64-encoded-JPEG, and selected that renderer first. It was returned the HTML-rendered JPEG image data, together with the String data-type object that could not be rendered. The system them passed the String object to the String renderer in order to retrieve the HTML representing the image description. In this way, every BioMoby data-type can be rendered by Gbrowse Moby.

**Figure 2:**
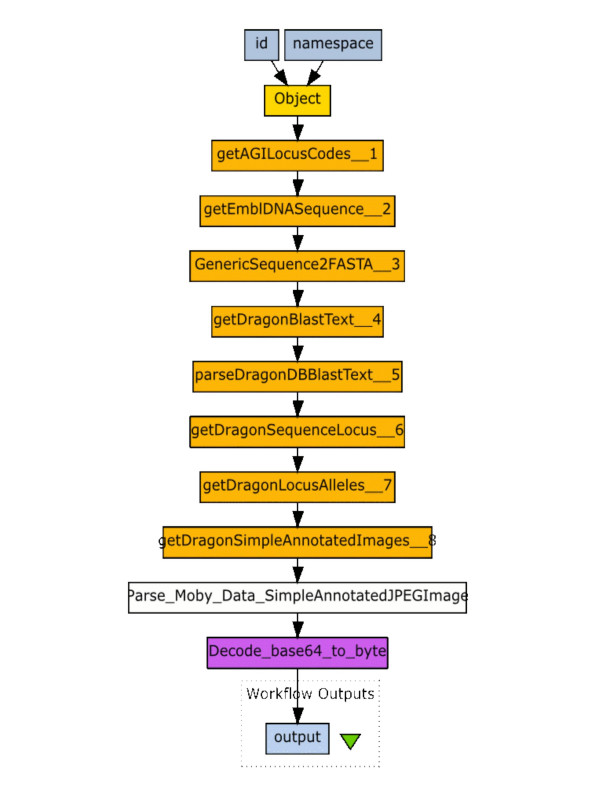
**A SCUFL workflow generated by Gbrowse Moby as displayed in the Taverna environment**. The workflow generated by following the example workflow in Table 1 was retrieved at step 17 and loaded into Taverna. The image shown here was exported from Taverna's wokflow overview screen. Note that Gbrowse Moby detected that the final data-type was a binary data-type in the BioMoby system (SimpleAnnotatedJPEGImage), and automatically added a parser and base-64 decoder to the last steps in the workflow such that the binary image would be properly displayed in the Taverna environment.

Retrieving the SCUFL document at Step 17 provides the workflow diagrammed in Figure 2 when loaded into Taverna, and will execute the workflow with high-throughput, following all possible links iteratively. The essence of this workflow is to look at the phenotypes of Snapdragon mutants whose affected locus shares homology with an Arabidopsis mutant whose phenotypic description includes a certain keyword – a process that would be extremely difficult in the absence of a workflow tool. However, the simplicity of the Gbrowse Moby interface makes it possible for a relatively naïve user to construct this workflow in just a few minutes.

## Conclusion

Gbrowse Moby was the first client capable of engaging BioMoby services, and has developed into a convenient yet powerful portal for "surfing" biological data. Though Gbrowse Moby itself is not capable of high-throughput analyses, it functions well as an environment for simultaneously designing and testing analytical pipelines that might then be utilized in workflow management environments such as Taverna [11], MOWserv [13], or Remora [14]; moreover, the ability to immediately access the SCUFL workflow description of your browsing session and load it into such a tool allows you to immediately move from a simple browsing environment to a high-throughput environment with little or no additional effort. Most importantly, however, Gbrowse Moby facilitates and guides the manual exploration of a global data-space, and the creation and immediate testing of multi-step analytical pipelines by non-informaticians, without requiring them to manipulate data formats, learn new web interfaces, or even have prior knowledge of the existence of the datasets and/or tools that they are engaging.

The power of having a common interface into a potentially unlimited number of data and analysis services cannot be underestimated, and this has been achieved thanks to community adoption of an end-user extensible ontological standard. Gbrowse Moby is one of the first examples of a semantically enabled bioinformatics data browser, and reveals an early glimpse of the power that Semantic Web Services will provide in the future.

## Availability and requirements

**Project name: **Gbrowse_moby

**Project home page: **A sub-package within the Generic Model Organism Database [9], or for personal use or proxy use over the Web [15].

**Operating systems(s): **Platform independent

**Programming Language: **Perl

**Other Requirements: **any CGI-capable webserver

**License: **Perl Artistic License

**Restrictions: **None

## Appendix 1

Resolve the LSID urn:lsid:biomoby.org:objectclass:SimpleAnnotatedJPEGImage:2001-09-21T16-00-00Z to metadata [16] to see the Web Ontology Language (OWL) class definition for SimpleAnnotatedJPEGImage.
